# *Hibiscus syriacus* L. Exhibits Cardioprotective Activity via Anti-Inflammatory and Antioxidant Mechanisms in an In Vitro Model of Heart Failure

**DOI:** 10.3390/life15081229

**Published:** 2025-08-03

**Authors:** Hung-Hsin Chao, Tzu-Hurng Cheng, Chun-Chao Chen, Ju-Chi Liu, Jin-Jer Chen, Li-Chin Sung

**Affiliations:** 1 Division of Cardiovascular Surgery, Department of Surgery, Shin Kong Wu Ho-Su Memorial Hospital, Taipei City 111045, Taiwan; m000675@ms.skh.org.tw; 2 School of Medicine, College of Medicine, Catholic Fu-Jen University, New Taipei City 24352, Taiwan; 3 Department of Biochemistry, School of Medicine, College of Medicine, China Medical University, Taichung City 404328, Taiwan; thcheng@mail.cmu.edu.tw; 4 Division of Cardiology, Department of Internal Medicine, Shuang Ho Hospital, Taipei Medical University, New Taipei City 23561, Taiwan; b101092035@tmu.edu.tw (C.-C.C.); liumdcv@tmu.edu.tw (J.-C.L.); 5 Division of Cardiology, Department of Internal Medicine, School of Medicine, College of Medicine, Taipei Medical University, Taipei City 11002, Taiwan; 6 Division of Cardiology, Department of Internal Medicine and Graduate Institute of Clinical Medical Science, China Medical University Hospital, Taichung City 404327, Taiwan; jc8510@yahoo.com; 7 Institute of Biomedical Sciences, Academia Sinica, Taipei City 11529, Taiwan

**Keywords:** heart failure, *Hibiscus syriacus* L., myocardial protection, reactive oxygen species, cytokine, inflammation

## Abstract

*Hibiscus syriacus* L. (HS), native to Eastern and Southern Asia, has been traditionally used in Asian herbal medicine for its anticancer, antimicrobial, and anti-inflammatory properties. Despite these recognized bioactivities, its potential cardioprotective effects, particularly in the setting of heart failure (HF), remain largely unexplored. This study aimed to investigate the effects of HS extracts and its bioactive constituents on angiotensin II (Ang II)-induced cardiac injury using an in vitro model with H9c2 rat cardiomyocytes. Cells exposed to Ang II were pretreated with HS extracts, and assays were performed to assess cell viability, reactive oxygen species (ROS) generation, protein synthesis, and secretion of inflammatory mediators, including tumor necrosis factor-alpha, interleukin 1β (IL-1β), and interleukin 6 (IL-6), as well as chemokine (CCL20) and HF-related biomarkers, such as brain natriuretic peptide (BNP) and endothelin-1. The results demonstrated that HS extracts significantly and dose-dependently attenuated Ang II-induced ROS accumulation and suppressed the secretion of pro-inflammatory cytokines, chemokines, BNP, and endothelin-1. Additionally, HS and its purified components inhibited Ang II-induced protein synthesis, indicating anti-hypertrophic effects. Collectively, these findings highlight the antioxidative, anti-inflammatory, and antihypertrophic properties of HS in the context of Ang II-induced cardiac injury, suggesting that HS may represent a promising adjunctive therapeutic candidate for HF management. Further in vivo studies and mechanistic investigations are warranted to validate its clinical potential.

## 1. Introduction

### 1.1. Heart Failure: Global Burden and Therapeutic Challenges

Heart failure (HF) is a major global health concern, characterized by impaired myocardial function that contributes to elevated morbidity and mortality [1,2]. Its prevalence is rising due to aging populations and increases in cardiovascular risk factors [2,3,4,5,6]. Beyond clinical impact, HF imposes significant economic burdens, highlighting the urgent need for novel, effective, and well-tolerated therapeutic strategies [7]. Although current first-line therapies such as renin–angiotensin–aldosterone system (RAAS) inhibitors and β-blockers offer symptomatic relief, their benefits are often constrained by adverse effects, including hyperkalemia and hypotension [8]. Moreover, conventional therapeutic approaches often lack efficacy in modulating the core pathophysiological processes that drive the progression of HF [8,9,10]. This underscores a critical need for alternative or adjunctive approaches that address both the symptomatic and mechanistic aspects of the disease.

### 1.2. Natural Products in Cardiovascular Therapy: A Focus on Hibiscus syriacus L.

**Figure 1:**
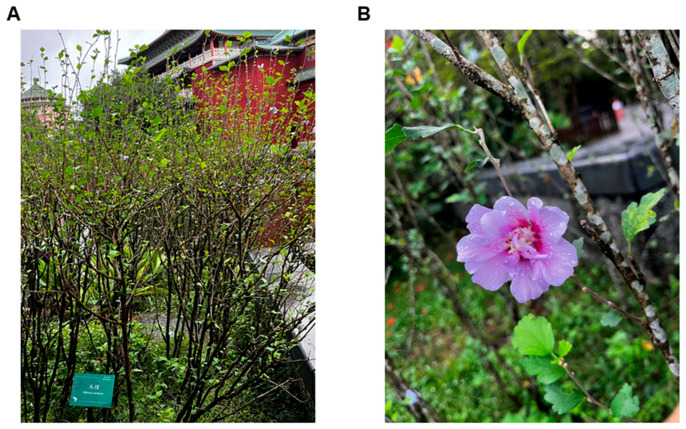
Photographs of *Hibiscus syriacus* L. This figure displays the distinctive features of *Hibiscus syriacus* L. (**A**) Complete morphology of the plant, including the stem, flowers, and leaves. (**B**) Close-up view of the plant’s characteristic flowers.

Natural compounds derived from medicinal plants have garnered increasing attention as complementary therapeutic agents, offering multitarget efficacy with favorable safety profiles [11,12,13,14,15]. In particular, *Hibiscus* spp. have been associated with antihypertensive, antioxidant, and anti-inflammatory effects [13]. Among them, *Hibiscus syriacus* L. (HS), commonly known as the Rose of Sharon (Figure 1), has a long history of use in traditional Asian medicine for a wide range of indications, including cardiovascular disorders, abdominal pain, skin diseases, and microbial infections [14,15,16]. Pharmacological studies have revealed that HS exerts antioxidative, anti-inflammatory, and vasodilatory effects (Table 1). Its bioactive constituents, such as flavonoids, phenolic acids, and anthocyanins, are known to modulate key pathological processes implicated in HF, including reactive oxygen species (ROS) production, inflammatory signaling, endothelial dysfunction, and myocardial remodeling [3,4,5,14,15,16,17]. In vitro evidence further supports its broader therapeutic applications, demonstrating anticancer, anti-osteoporotic, and sleep-improving properties via mechanisms involving Notch, GSK-3β, and autophagy signaling [3,4,15,18,19]. These findings position HS as a promising botanical candidate for cardiovascular protection, particularly in HF.

### 1.3. Rationale, Research Gaps, and Study Objectives

Despite encouraging preclinical data, the therapeutic potential of HS in the context of HF remains underexplored, with limited understanding of its molecular mechanisms and clinical applicability [29,30]. There is a need to delineate how HS modulates key HF-related pathways and biomarkers, including oxidative stress and proinflammatory mediators such as tumor necrosis factor-alpha (TNF-α), interleukin-1β (IL-1β), interleukin-6 (IL-6), and C-C motif chemokine ligand 20 (CCL20), as well as brain natriuretic peptide (BNP) and endothelin-1 (ET-1) [18,21,31,32,33]. This study aims to evaluate the cardioprotective effects of HS extracts using an in vitro model, focusing on its antioxidant and anti-inflammatory activities. By constructing a conceptual framework around HS as a novel plant-based intervention for HF, this research provides a foundational step toward future translational and clinical development.

## 2. Materials and Methods

### 2.1. Selection and Preparation of HS Extracts

The flowers and stems of HS were collected from Beitun District, Taichung City, Taiwan. Botanical identification was performed by a qualified taxonomist based on morphological characteristics and regional floristic references. Although a voucher specimen was not deposited, the botanical identity and consistency of the plant material were verified through visual inspection and taxonomic assessment, in accordance with established practices [5,15]. To prepare the extracts, both maceration and Soxhlet methods were employed to optimize yield and preserve bioactivity. Air-dried and pulverized HS flowers and stems were first macerated in 95% ethanol at ambient temperature (22–25 °C) for 72 h with intermittent agitation. The resulting ethanol filtrate was concentrated under reduced pressure at 40 °C to produce the crude ethanol extract (HS-EtOH). This extract was subsequently partitioned using solvents of increasing polarity—hexane (HS-Hex), ethyl acetate (HS-EA), n-butanol (HS-BuOH), and distilled water (HS-H_2_O)—to fractionate compounds based on solubility and polarity, in accordance with established phytochemical fractionation protocols [18,21]. In parallel, Soxhlet extraction was conducted using 10 g of dried HS stem powder for each solvent. Extraction was performed over 6 h at controlled temperatures specific to each solvent: 60 °C for ethanol, 50 °C for hexane, 55 °C for ethyl acetate, and 65 °C for n-butanol. Aqueous extraction was achieved through refluxing in distilled water at 90 °C for 2 h. All extracts were subsequently filtered, concentrated using rotary evaporation, and stored at −20 °C until further use. Extraction yields were calculated relative to the initial dry weight of the plant material. Preliminary phytochemical analysis confirmed the presence of key secondary metabolites, including flavonoids, anthocyanins, and polyphenols, consistent with previously reported profiles of HS extracts [23,28]. This integrative and stepwise extraction strategy was designed to maintain the stability of thermolabile compounds and to enrich fractions with potential antioxidant and anti-inflammatory activities, as supported by earlier investigations into the pharmacological properties of HS-derived constituents [19,22].

### 2.2. Cell Viability Test

Cell viability was evaluated using the Cell Counting Kit-8 assay to determine the cytotoxic threshold and optimal concentration range of HS extract for subsequent mechanistic studies. H9c2 cells were seeded into 96-well plates at a density of 8 × 10^3^ cells per well and incubated for 24 h to allow cell attachment. Thereafter, cells were treated with varying concentrations of HS extracts (10, 30, 50, 100, 150, and 200 μg/mL) for an additional 24 h. Following treatment, 10 μL of WST-8 reagent [2-(2-methoxy-4-nitrophenyl)-3-(4-nitrophenyl)-5-(2,4-disulfophenyl)-2H-tetrazolium, monosodium salt] was added to each well, and plates were incubated at 37 °C for 4 h. The absorbance was measured at 450 nm using a microplate reader (Bio-Rad, Hercules, CA, USA), and cell viability was calculated relative to untreated control wells.

### 2.3. Experimental Design for Assessing ROS Production

To explore the role of oxidative stress in HS-mediated cardioprotection, ROS levels were quantified in H9c2 cells following Ang II stimulation and HS pretreatment.

#### 2.3.1. Materials

Tissue culture reagents, including fetal bovine serum and Dulbecco’s modified Eagle’s medium (DMEM), were obtained from Invitrogen (Carlsbad, CA, USA). The ROS-sensitive fluorescent dye, 2′,7′-dichlorofluorescin diacetate (DCFH-DA), was procured from Serva Co. (Heidelberg, Germany). Enzyme-linked immunosorbent assay (ELISA) kits were purchased from Abcam (Cambridge, UK).

#### 2.3.2. H9c2 Cell Culture

The H9c2 cell line, an immortalized rat embryonic cardiac myoblast line, was sourced from the American Type Culture Collection (ATCC CRL-1446; Rockville, MD, USA). The characteristics of this cell line have been previously described [34]. The cells were cultured in DMEM supplemented with 10% fetal bovine serum, all purchased from Sigma-Aldrich. The cell cultures were maintained in a humidified incubator at 37 °C with 5% CO_2_.

#### 2.3.3. Flow Cytometric Assay of 2′,7′-Dichlorodihydrofluorescein Oxidation

To assess intracellular ROS levels, cells were incubated in culture medium containing 30 µM DCFH-DA for 1 h prior to treatment with HS or Ang II (100 nM), following established protocols [35,36]. The same concentration of DCFH-DA was maintained during treatment. After incubation, cells were washed with phosphate-buffered saline (PBS), detached via brief trypsinization, and analyzed for DCF fluorescence. DCFH-DA is hydrolyzed by cellular esterases to DCFH, which is oxidized to fluorescent DCF in the presence of ROS. The fluorescence intensity of DCF, indicative of intracellular ROS levels, was measured using a fluorescence spectrophotometer, with excitation and emission wavelengths set at 475 and 525 nm, respectively. Following fluorescence measurements, cell counts were determined using an automated cell counter (S.ST.II/ZM, Coulter Electronics Ltd., Miami, FL, USA), and ROS levels were normalized to cell number. This assay provided mechanistic insight into whether HS exerts antioxidant effects in Ang II-stimulated cardiomyocytes, a known contributor to HF pathogenesis.

### 2.4. Experimental Design for Assessing Ang II-Stimulated Protein Synthesis

To further delineate whether the antioxidant effect of HS translates into functional protection, we next assessed cardiomyocyte hypertrophy by measuring protein synthesis. Protein synthesis was evaluated by measuring the incorporation of [^3^H]leucine into acid-insoluble protein, a validated marker of hypertrophic response [37]. Cardiomyocytes were incubated in serum-free medium containing 1.0 μCi/mL [^3^H]leucine. Following incubation, the cells were harvested by treatment with 5% trichloroacetic acid at 4 °C and then solubilized in 0.1 N NaOH. The rate of [^3^H]leucine incorporation was quantified using liquid scintillation spectrometry. This experiment aimed to investigate whether the modulation of ROS by HS is associated with downstream changes in hypertrophic signaling.

### 2.5. Experimental Design for Assessing the Secretion of the Selected Inflammatory Cytokines, Chemokines, and HF Risk Biomarkers

Given the key role of inflammation in HF, we subsequently examined the effects of HS on the secretion of inflammatory cytokines and HF-associated biomarkers. H9c2 cells were pretreated with or without HS extracts or its bioactive components for 12 h, followed by stimulation with Ang II (100 nM) for 24 h. After centrifugation at 1000 rpm for 10 min, supernatants were collected to measure levels of TNF-α, IL-1β, IL-6, CCL20, BNP, and ET-1. These biomarkers were quantified using commercially available ELISA kits (Abcam, Cambridge, UK), following the manufacturer’s instructions [38]. These measurements were intended to confirm whether HS attenuates inflammatory and neurohormonal responses associated with HF pathophysiology.

## 3. Results

### 3.1. HS Extractions

#### 3.1.1. Bioactive Compounds and Their Therapeutic Potential

**Figure 2:**
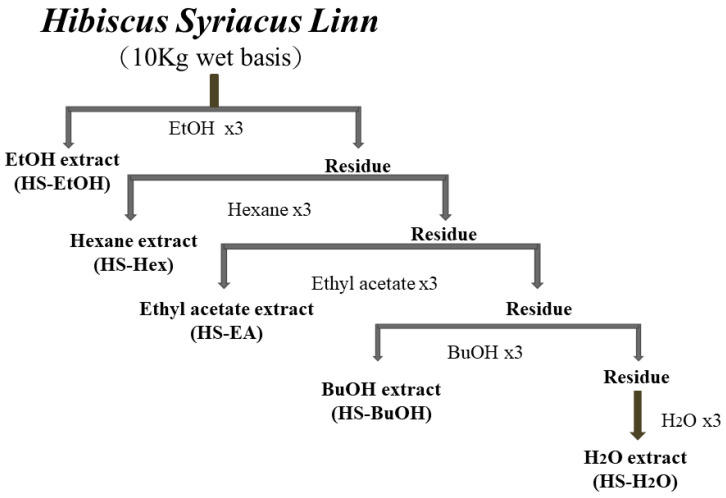
Preparation and Sequential Fractionation of Crude *Hibiscus syriacus* L. Extracts. This figure illustrates the stepwise extraction and fractionation protocol used to isolate bioactive constituents from *Hibiscus syriacus* L. (HS) extracts. Initially, the plant material was extracted three times with 95% ethanol, chosen for its broad-spectrum solubility and ability to recover both polar and non-polar compounds, including flavonoids, sterols, and anthocyanins [5,26]. The combined ethanol extracts were concentrated under reduced pressure to yield the crude ethanol extract (HS-EtOH). To enrich specific phytochemical classes, HS-EtOH was sequentially partitioned using solvents of increasing polarity—hexane (HS-Hex), ethyl acetate (HS-EA), n-butanol (HS-BuOH), and water (HS-H_2_O)—based on solubility gradients [18,21]. This polarity-guided fractionation enabled the separation of structurally diverse compounds, facilitating targeted analysis of antioxidant, anti-inflammatory, and cytotoxic activities reported in previous studies [15,24,27]. Adapted from the master’s thesis of Wei-Ping Chiang [39]. The resulting fractions were used for phytochemical profiling and bioactivity assays.

**Figure 3:**
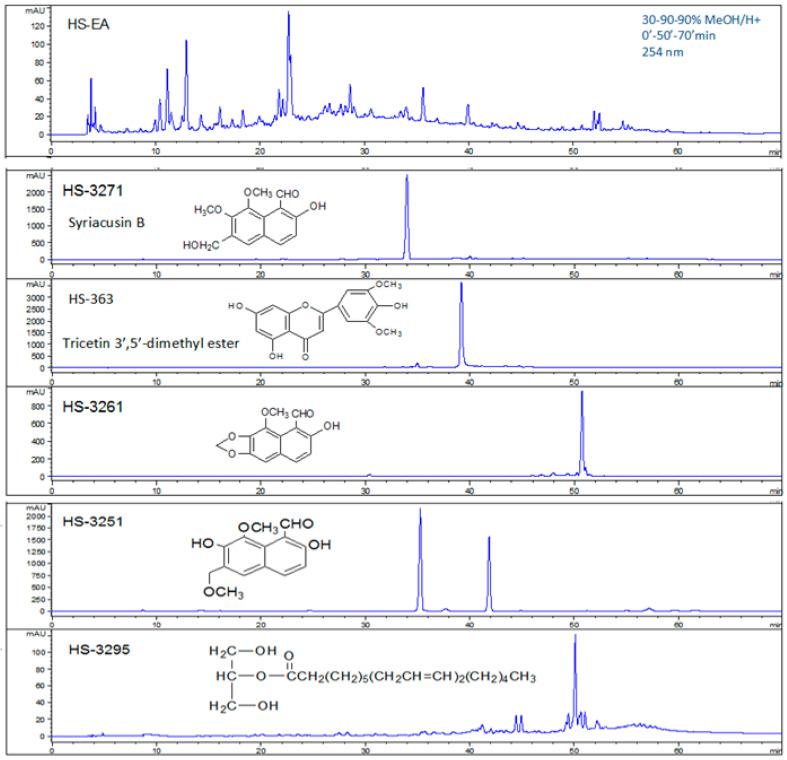
High-Performance Liquid Chromatography (HPLC) and Nuclear Magnetic Resonance (NMR) Analysis. This figure presents the analytical methods used to identify and characterize bioactive compounds extracted from HS. HPLC was used to separate and quantify phytochemical constituents, enabling the identification of specific compounds such as Syriacusin B (HS-3271), linoleic acid (HS-3253) and 2-linoleodistearin (HS-3295). NMR spectroscopy was subsequently used to determine the molecular structures and functional groups of these isolates, confirming the presence of key bioactive compounds, including syriacusin B and tricetin 3′,5′-dimethyl ester. Adapted from the master’s thesis of Wei-Ping Chiang [39]. These analyses verified compound purity and chemical identity, providing molecular validation for downstream biological testing.

To explore the pharmacological potential of HS extraction, we first characterized its chemical composition. To maximize the recovery of bioactive constituents from HS, an ethanol-first extraction strategy was employed. Ethanol was selected as the initial solvent due to its broad-spectrum solubility, which enables efficient extraction of both polar and non-polar phytochemicals, including flavonoids, anthocyanins, sterols, and polyphenols [5,26]. This approach is consistent with previous studies demonstrating the pharmacological relevance of ethanol-based extracts in antioxidant, anti-inflammatory, and cytotoxic applications [15,21]. Following ethanol maceration, the crude extract (HS-EtOH) was subjected to sequential solvent partitioning using hexane (HS-Hex), ethyl acetate (HS-EA), n-butanol (HS-BuOH), and distilled water (HS-H_2_O), allowing for polarity-based fractionation (Figure 2). This stepwise method was designed to isolate distinct classes of compounds, as previously described in studies investigating HS-derived anthocyanins, sterols, and triterpenoids with diverse bioactivities [22,27]. The extraction yields, calculated based on dry weight, were as follows: HS-Hex (2.3%), HS-EA (4.7%), HS-BuOH (6.1%), and HS-H_2_O (8.4%). These values reflect solvent-specific recovery efficiencies and align with prior reports on HS phytochemical distribution [17,28]. Fourier-transform infrared spectroscopy confirmed the presence of functional groups characteristic of phenolic acids, sterols, and flavonoids. Specific compounds, including β-sitosterol, 2-linoleodistearin, linoleic acid, syriacusin B, and tricetin 3′,5′-dimethyl ester, were identified using high-performance liquid chromatography (HPLC) and nuclear magnetic resonance (NMR) spectroscopy (Figure 3). HPLC analysis was performed using a reverse-phase C18 column (250 mm × 4.6 mm, 5 µm particle size), with a gradient mobile phase consisting of methanol and water (0.1% formic acid), at a flow rate of 1.0 mL/min and detection wavelength of 254 nm. Elution profiles were monitored over a 30 min run time. Structural elucidation of isolated compounds was confirmed by ^1^H and ^13^C NMR spectroscopy using a Bruker 500 MHz instrument in CDCl_3_ or DMSO-d_6_ as solvents, with chemical shifts referenced to internal TMS. These spectral data supported the identification of key bioactive constituents previously associated with antioxidant, anti-inflammatory, and cytotoxic activities in HS [15,22,27]. Collectively, these findings supported the therapeutic relevance of HS as a natural source of multifunctional bioactive compounds, with potential applications in cardiovascular, dermatological, and metabolic health.

#### 3.1.2. Effects of HS Extracts on H9c2 Cardiomyocyte Viability

**Figure 4:**
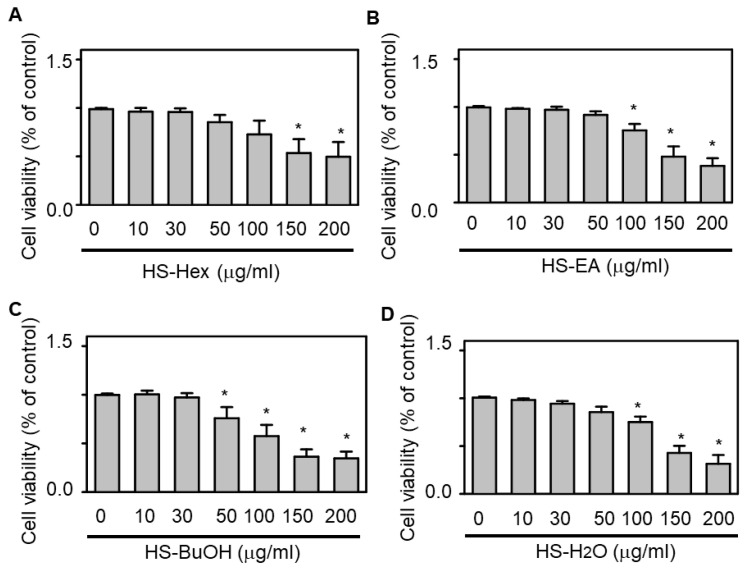
Cytotoxicity of HS Extracts Against H9c2 Cells. Cell viability was evaluated using the Cell Counting Kit-8 following exposure to varying concentrations of HS extracts: HS-Hex (panel **A**), HS-EA (panel **B**), HS-BuOH (panel **C**), and HS-H_2_O (panel **D**). Untreated H9c2 cells served as the control, with viability normalized to 100%. The results are presented as mean ± standard error of the mean (SEM; *n* = 6). Statistically significant differences were observed for comparisons with untreated controls (* *p* < 0.05). This experiment was conducted to determine non-toxic concentration ranges for use in subsequent mechanistic assays.

To determine the non-toxic range for downstream functional assays, the treatment of cultured H9c2 cardiomyocytes with HS extracts at concentrations of 10–50 µg/mL for 24 h did not significantly affect cell viability (HS-Hex, HS-EA, and HS-H_2_O; Figure 4).

### 3.2. Effects of HS Extracts on ROS Production, Protein Synthesis, and Secretion of Inflammatory Cytokines, Chemokines, and HF Risk Biomarkers

#### 3.2.1. Inhibition of Ang II-Induced Redox Signaling and Hypertrophy In Vitro

**Figure 5:**
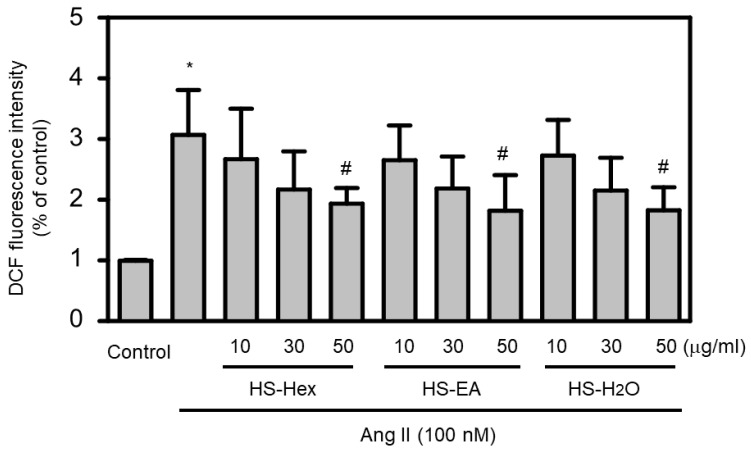
Effects of HS Extracts on Ang II-Stimulated ROS Production in H9c2 Rat Cardiomyocytes. H9c2 cells were pretreated with vehicle control or varying concentrations of HS extracts (HS-Hex, HS-EA, and HS-H_2_O; 10, 30, and 50 μg/mL) for 24 h, followed by cotreatment with Ang II (100 nM) for 2 h. ROS production was measured using DCF-based fluorescence assays, with fluorescence intensities in vehicle-treated control cells normalized to 100%. The results are expressed as mean ± SEM (*n* = 5). Statistical analysis revealed significant differences: * *p* < 0.05 compared with untreated controls; ^#^ *p* < 0.05 compared with cells treated with Ang II alone. This assay assessed the antioxidant potential of HS extracts in an oxidative stress model relevant to HF.

**Figure 6:**
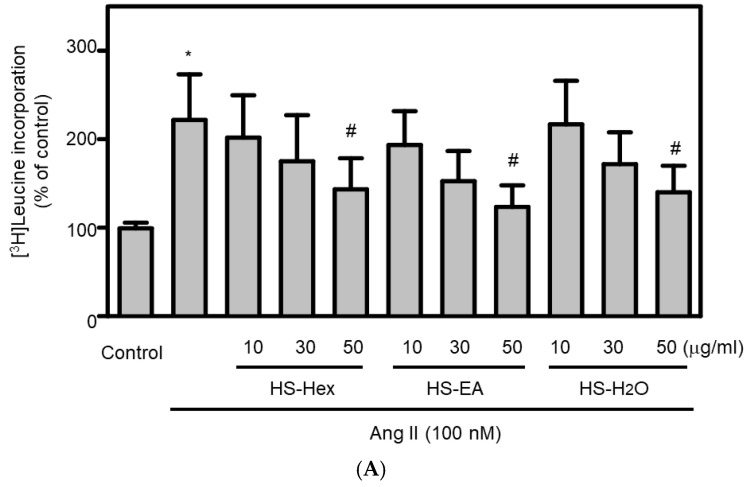

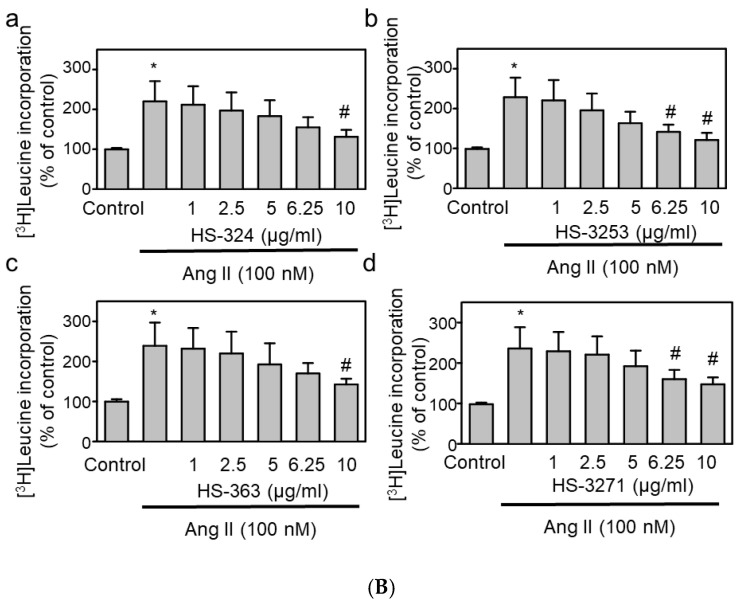
(**A**)**.** Effects of HS Extracts on Ang II-Stimulated Protein Synthesis in H9c2 Rat Cardiomyocytes. H9c2 rat cardiomyocytes were pretreated with either a vehicle control or various concentrations of HS extracts (HS-Hex, HS-EA, and HS-H_2_O; 10, 30, and 50 μg/mL) for 24 h, followed by cotreatment with Ang II (100 nM) for an additional 24 h. The results are presented as mean ± SEM, based on triplicate determinations across each experiment (*n* = 5). Significant differences were observed: * *p* < 0.05 compared with vehicle control; ^#^ *p* < 0.05 compared with Ang II treatment alone. This experiment evaluated the potential of HS extracts to prevent cardiomyocyte hypertrophy, a hallmark of HF. (**B**). Effects of Pure Compounds on Ang II-Stimulated Protein Synthesis in H9c2 Rat Cardiomyocytes. H9c2 rat cardiomyocytes were pretreated with either a vehicle control or varying concentrations of pure compounds extracted from HS—HS-324 (**a**), HS-3253 (**b**), HS-363 (**c**), and HS-3271 (**d**)—for 24 h, followed by cotreatment with Ang II (100 nM) for an additional 24 h. The results are presented as the mean ± SEM for each experiment (*n* = 5), with triplicate determinations. Significant differences were noted: * *p* < 0.05 compared with vehicle control; ^#^ *p* < 0.05 compared with Ang II treatment alone. This figure confirms that isolated bioactive compounds from HS also attenuate Ang II-induced hypertrophic signaling.

We next examined the antioxidant capacity of HS extracts in an in vitro model of Ang II-induced oxidative stress. Treatment of cardiomyocytes with HS extracts resulted in a concentration-dependent reduction in Ang II-increased ROS levels compared with Ang II-treated controls (Figure 5). Notably, higher concentrations of HS extracts significantly attenuated ROS production, indicating their antioxidant activity. Statistical analysis confirmed a clear dose-dependent relationship between HS extract concentration and ROS inhibition. These findings suggested that HS extracts may possess antioxidant properties that contribute to their cardioprotective effects in HF. Given the established role of ROS in promoting hypertrophy, cardiomyocyte hypertrophy, a hallmark of HF, is characterized by increased protein synthesis. In this study, cardiomyocytes treated with Ang II (100 nM, 24 h) exhibited increased protein synthesis compared with control cells (Figure 6A). To investigate the antihypertrophic effects of HS extracts, cardiomyocytes were treated with various HS extracts, including HS-Hex, HS-EA, and HS-H_2_O. This treatment significantly reduced Ang II-induced protein synthesis compared with cells treated with Ang II alone (Figure 6A). Moreover, cardiomyocytes pretreated with pure compounds isolated from HS extracts (HS-324, HS-3253, HS-363, and HS-3271 at 6.25 and 10 μM) for 12 h did not exhibit an increase in Ang II-induced protein synthesis, in contrast to the Ang II-stimulated control groups (Figure 6B). These results indicated that pretreatment with HS extracts or their isolated components may effectively prevent Ang II-induced cardiomyocyte hypertrophy.

#### 3.2.2. Inhibition of Ang II-Induced Inflammatory Cytokine and Chemokine Secretion In Vitro

**Figure 7:**
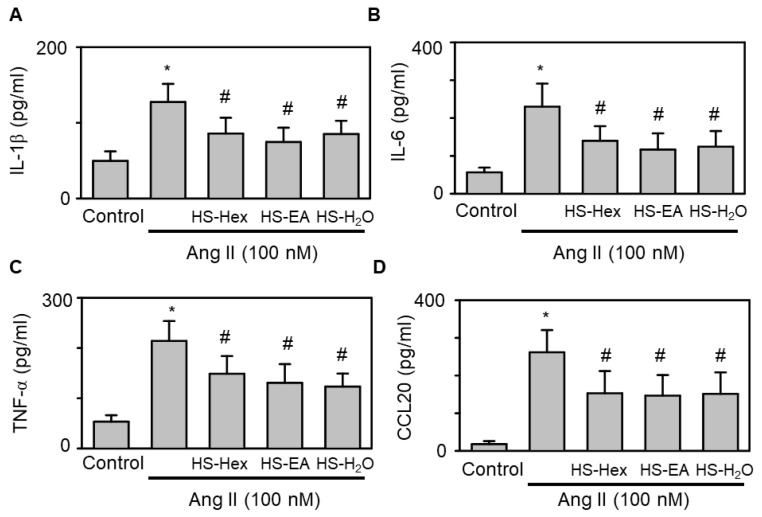
Effects of HS Extracts on Ang II-Stimulated Inflammatory Cytokines and Chemokines in H9c2 Rat Cardiomyocytes. The expression levels of inflammatory biomarkers, including interleukin IL-1β (IL-1β; panel **A**), interleukin 6 (IL-6; panel **B**), tumor necrosis factor-α (TNF-α; panel **C**), and CC chemokine ligand 20 (CCL20; panel **D**), were assessed. H9c2 cells were pretreated with a vehicle control or various HS extracts (HS-Hex, HS-EA, and HS-H_2_O; 50 μg/mL) for 24 h, followed by cotreatment with Ang II (100 nM) for an additional 24 h. The results are presented as mean ± SEM (*n* = 5). Statistical analysis revealed significant differences: * *p* < 0.05 compared with untreated controls; ^#^ *p* < 0.05 compared with cells treated with Ang II alone. This experiment demonstrated the anti-inflammatory activity of HS extracts in an in vitro HF model.

As inflammation is a major contributor to HF progression, we next assessed the anti-inflammatory potential of HS extracts. As demonstrated in Figure 7, pretreatment with HS extracts effectively inhibited the secretion of inflammatory cytokines and chemokines, including TNF-α, IL-1β, IL-6, and CCL20, in response to 2 h Ang II (100 nM) stimulation. This finding indicated that HS extracts exert anti-inflammatory effects by suppressing the Ang II-induced production of inflammatory mediators. Such effects may contribute to the potential cardioprotective benefits of HS in HF.

#### 3.2.3. Inhibition of Ang II-Induced HF Risk Biomarker Secretion In Vitro

**Figure 8:**
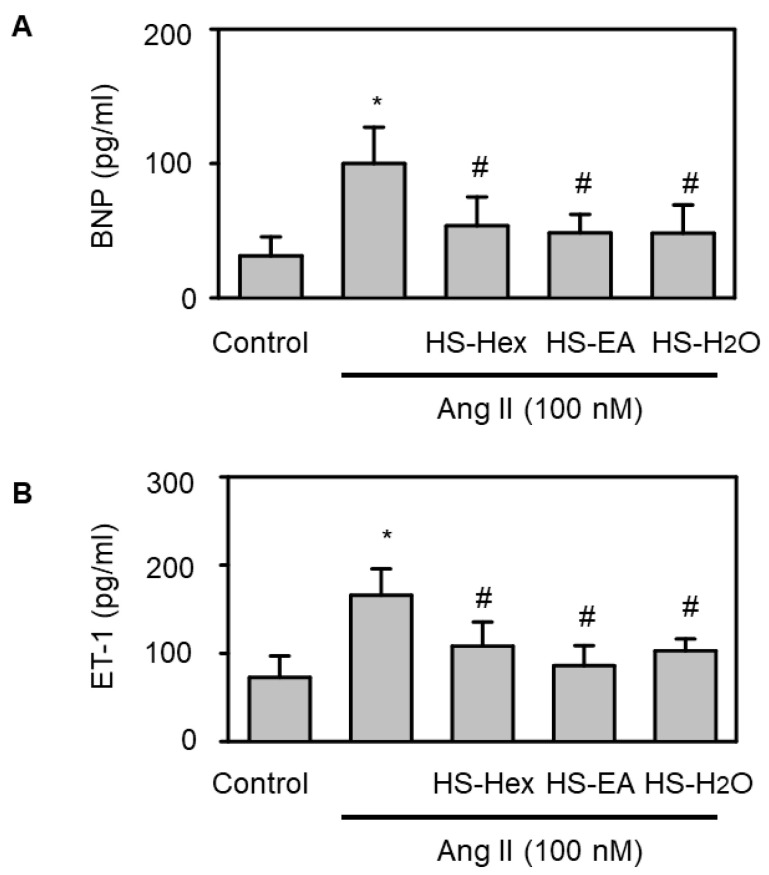
Effects of HS Extracts on Ang II-Stimulated Heart Failure Biomarkers in H9c2 Rat Cardiomyocytes. The levels of heart failure risk biomarkers, including brain natriuretic peptide (BNP; panel **A**) and endothelin-1 (ET-1; panel **B**), were analyzed. H9c2 cells were pretreated with vehicle or various HS extracts (HS-Hex, HS-EA, and HS-H_2_O; 50 μg/mL) for 24 h, followed by cotreatment with Ang II (100 nM) for an additional 24 h. The results are presented as mean ± SEM (*n* = 6). Statistical analysis revealed significant differences: * *p* < 0.05 compared with untreated controls; ^#^ *p* < 0.05 compared with cells treated with Ang II alone. These data support the potential of HS extracts in modulating neurohormonal markers associated with HF progression.

To evaluate whether HS extracts influence key markers of HF pathophysiology, we quantified the release of BNP and ET-1 following Ang II exposure. As illustrated in Figure 8, pretreatment with HS extracts significantly reduced the secretion of biomarkers for HF risk, including ET-1 and BNP, in response to a 2 h Ang II (100 nM) stimulation. This finding suggested that HS extracts can effectively suppress Ang II-induced production of HF risk biomarkers, further supporting their potential role as cardioprotective agents and underscoring their therapeutic relevance in HF management.

## 4. Discussion

### 4.1. Interpretation of Experimental Results and Mechanistic Interpretations

Inflammation and oxidative stress are pivotal contributors to the pathogenesis of HF [32,33]. The present findings highlight the therapeutic potential of HS extracts in HF management. The observed reduction in ROS production reflects the potent antioxidant capacity of HS, which may help to alleviate oxidative stress—a hallmark of HF [20,21]. HS extracts also significantly suppressed the secretion of inflammatory cytokines in a dose-dependent manner, further reinforcing their cardioprotective potential [22]. Excessive ROS generation can lead to DNA damage, lipid peroxidation, and cell death, thereby promoting myocardial remodeling and HF progression [35,40]. Inflammation is also widely recognized as a central pathophysiological factor in both acute and chronic HF and serves as an independent predictor of poor prognosis, regardless of left ventricular systolic function [41,42]. Elevated levels of inflammatory cytokines are strongly associated with increased HF severity [42,43], with TNF-α, IL-1, IL-6, IL-8, IL-10, IL-18, and IL-33 identified as key mediators in HF pathogenesis [32,42,43]. Notably, TNF-α has been strongly implicated in cardiac inflammation and correlates with both HF severity and mortality when serum levels are elevated [44]. Chemokines, which mediate immunoregulatory and inflammatory responses, also contribute to HF progression [45]. Serum CCL20 levels have been associated with ischemic heart disease [46] and are considered potential predictors of HF severity and prognosis [44]. Furthermore, TNF-α-induced upregulation of CCL20 expression in human cardiac fibroblasts suggests a potential pathogenic link between these molecules in cardiac inflammation [44]. Biomarkers such as BNP, ET-1, hsTn1, and ST2 are widely used to assess HF risk and prognosis [31]. Elevated discharge levels of these biomarkers in HF patients are associated with increased risks of readmission and mortality compared with baseline values.

Our findings are consistent with those of studies identifying oxidative stress and inflammation as critical therapeutic targets in HF [18]. The dose-dependent effects observed in our study suggest that HS modulates key cellular pathways involved in HF pathophysiology. Future investigations are warranted to further elucidate the precise molecular mechanisms underlying these cardioprotective effects.

### 4.2. Comparison with Previous Studies

Our results align with previous findings that underscore the therapeutic potential of HS extracts [18,21]. In BV2 microglia cells, anthocyanins isolated from HS petals exhibited strong anti-inflammatory and antioxidant effects by attenuating endoplasmic reticulum stress and NF-κB signaling, reducing mitochondrial ROS production, and suppressing NLRP3 inflammasome-mediated IL-1β and IL-18 secretion [23]. Similarly, in H_2_O_2_-stimulated HaCaT keratinocytes, these anthocyanins activated the nuclear factor erythroid 2-related factor 2 (Nrf2) and heme oxygenase-1 pathways, conferring cytoprotective effects [21]. In lipopolysaccharide (LPS)-stimulated RAW 264.7 macrophages, HS-derived anthocyanins have also been shown to downregulate inducible nitric oxide synthase (iNOS) and cyclooxygenase-2 (COX-2), reduce pro-inflammatory cytokine levels (e.g., TNF-α, IL-6, and IL-12), and inhibit NF-κB activation [22]. Studies on Ang II signaling pathways in cardiomyocytes have identified key pathways contributing to HF progression, including redox-sensitive ERK1/2, p38, and NF-κB pathways driving Ang II-induced ROS production [47], ER stress triggered by excessive ROS [48], and activation of the NLRP3 inflammasome leading to elevated IL-1β and IL-18 levels [49]. Our findings extend this growing body of evidence by providing mechanistic insights into HS-mediated cardioprotection. Specifically, the data support the antioxidant and anti-inflammatory actions of HS, contributing to the attenuation of HF-related pathologies. This study broadens the current understanding of the molecular targets of HS and supports its development as a natural therapeutic candidate for HF management.

### 4.3. Implications of Findings in the Context of HF Management

**Figure 9:**
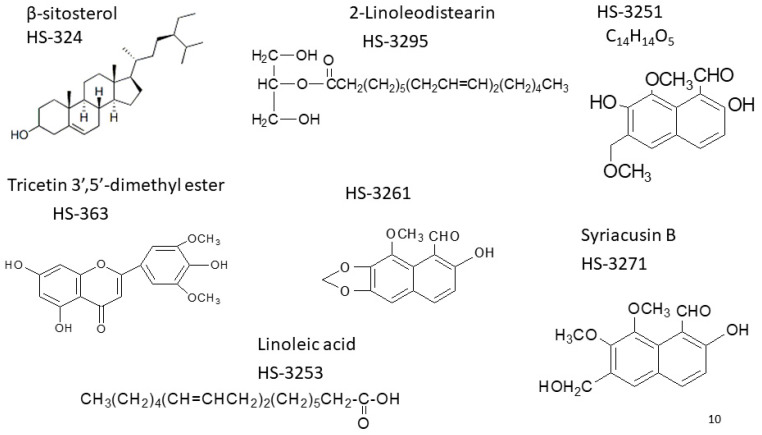
Molecular Structures of Pure Compounds Isolated from HS Extracts. The figure displays the molecular structures of several pure compounds isolated from HS extracts, including β-sitosterol (HS-324), linoleic acid (HS-3253), 2-linoleodistearin (HS-3295), and syriacusin B (HS-3271). Adapted from the master’s thesis of Wei-Ping Chiang [39]. These structures confirm the chemical identities of isolated compounds used in functional assays.

The present findings suggest that HS extracts may attenuate HF progression through the reduction of oxidative stress, suppression of inflammatory responses, and limitation of myocardial remodeling [21]. Several key inflammatory mediators and HF biomarkers, including TNF-α, IL-1β, IL-6, and BNP, are known to be produced directly by cardiomyocytes [42,50]. The chemokine CCL20, initially identified in the liver, lung, and appendix, is now recognized as being expressed in various tissues and has been linked to ventricular dysfunction and the severity of ischemic heart disease [45,46,51]. ET-1, discovered over 30 years ago as the first endothelium-derived contracting factor, plays a key role in inflammatory signaling, particularly through interactions with TNF-α, IL-1, and IL-6 [52,53]. Cardiomyocytes both express endothelin receptors and synthesize ET-1 [53]. The autocrine and paracrine activity of ET-1 plays a crucial role in the development of cardiomyocyte hypertrophy [54]. TNF-α mediates several adverse effects on cardiac function and structure, including negative inotropic effects due to disrupted calcium homeostasis, the upregulation of other inflammatory molecules, and enhanced endothelial–leukocyte interactions [42]. IL-1β, a key driver of cardiac inflammation, promotes cardiomyocyte apoptosis and the recruitment of inflammatory cells to the myocardium [50]. IL-6 similarly impairs cardiac function by inducing negative inotropic effects and promoting cardiac hypertrophy and fibrosis [50]. Elevated IL-6 levels have been associated with diuretic resistance and poor clinical outcomes, including mortality in patients with HF [42,55]. In acute HF, IL-6 levels measured 48–72 h after admission were independently predictive of 30-day mortality [56], and patients with cardiogenic shock exhibited an early surge in IL-6 levels [57]. These findings suggest that HF progression is driven, in part, by sustained chronic inflammation in addition to neurohormonal activation [58]. Long-term pharmacological modulation of chronic inflammation remains challenging due to the associated adverse effects. Therefore, the present study emphasizes the therapeutic promise of natural compounds, such as HS, as adjunctive agents in HF management. Given the limitations of conventional therapies, HS represents a potentially safer and more cost-effective alternative, particularly for use in resource-limited settings. As mentioned, several bioactive compounds have been isolated from HS extracts—β-sitosterol (HS-324), linoleic acid (HS-3253), syriacusin B (HS-3271), and tricetin 3′,5′-dimethyl ester (HS-363)—each warranting further investigation for their therapeutic relevance in HF (Figure 9). β-Sitosterol, a phytosterol, has demonstrated multiple biological effects, including stabilization of the phospholipid bilayers of cell membranes, improved glycemic control in adipose tissue in high-fat diet-fed type-2 diabetic rats, and protection against hypoxia/reoxygenation-induced H9c2 cardiomyocyte injury through oxidative stress reduction and mitochondrial function enhancement [59,60,61]. Linoleic acid, a dietary omega-6 polyunsaturated fatty acid, has been inversely associated with cardiovascular risk and systemic inflammation in both dietary and circulating biomarker studies [62]. Tricetin 3′,5′-dimethyl ester, also known as tricin, has been demonstrated to attenuate oxidative stress and suppress inflammatory mediator expression in vivo, particularly in murine models of myocardial injury induced by coronary artery ligation [63]. Tricin’s molecular mechanisms include inhibition of iNOS, COX-2, and NF-κB signaling, further supporting the relevance of plant-derived polyphenols in cardiovascular oxidative stress modulation [64]. Syriacusin B, a naphthalene compound isolated from the methanol extract of HS, has demonstrated inhibitory effects on lipid peroxidation, highlighting its potential antioxidant properties [65,66]. In addition, the antioxidant activities exhibited by various parts of HS highlight the plant’s phytochemical diversity and functional potential. In radical scavenging assays, the 70% ethanol extract showed that petals had the highest antioxidant activity (56.4% at 25 μg/mL), followed by leaves (45.3%), sprouts (31.4%), and roots (25.4%)—a trend that correlated with the respective polyphenol content in each tissue [5]. Further studies are warranted to evaluate the clinical efficacy, safety profile, and optimal dosing strategies for HS-based interventions in HF. Such efforts will lay the groundwork for integrating HS-derived compounds into evidence-based clinical practice.

### 4.4. Limitations of the Study and Future Directions

This study presents several limitations that should be considered. The in vitro design, while informative for mechanistic insight, limits direct clinical applicability. The absence of in vivo validation precludes assessment of pharmacokinetics, tissue distribution, and systemic effects. Thus, animal models and clinical trials are necessary to confirm the translational relevance of HS in HF treatment. The investigation primarily focused on antioxidant and anti-inflammatory effects in cardiomyocytes, without addressing other critical HF mechanisms, such as fibrosis, apoptosis, or neurohormonal activation or mitochondrial dysfunction. Furthermore, although we proposed involvement of NF-κB, Nrf2/HO-1, and ERK1/2 signaling, these pathways were not directly verified. The lack of in silico modeling or pathway-specific assays limits mechanistic conclusiveness, highlighting a key direction for future research. Moreover, inter-assay and inter-laboratory variability in extraction protocols, solvent composition, and assay conditions may influence the reproducibility of results. Standardization of extraction and quantification procedures will be necessary to ensure consistency and comparability across future investigations. Additionally, prior studies have shown that higher concentrations of HS extracts may reduce immune cell viability, raising concerns about dose-dependent cytotoxicity across different cell types [22]. Future dose–response and cell-type specificity studies are necessary to address this concern. Lastly, the long-term safety and efficacy of HS supplementation remain unknown. Robust clinical investigations are essential to establish its pharmacological profile and determine its potential as an adjunctive therapy in HF.

## 5. Conclusions

This study demonstrated the therapeutic potential of HS extracts in HF management, with significant reductions in ROS production and inflammatory mediators, indicative of cardioprotective properties. By elucidating the antioxidant and anti-inflammatory mechanisms of HS, our findings deepen the understanding of HF pathophysiology and suggest novel avenues for treatment. Given the limitations of current therapies in managing chronic inflammation and oxidative stress, HS offers promise as an adjunctive or alternative strategy. Future research should prioritize rigorous clinical trials to validate the efficacy and safety of HS, optimize its dosing and formulations, and explore its potential synergistic effects with established HF therapies, thereby advancing the field of cardiovascular therapeutics.

## Figures and Tables

**Table 1:** Summary of reported biological activities of *Hibiscus syriacus* L.

Plant Part Used	Preparation Type	Reported Biological Activity	Reference
Callus culture	Extract	Cytotoxicity in colorectal cancer via Notch-mediated cholesterol biosynthesis suppression	Xu et al., 2022 [15]
	Gold nanoparticle biosynthesis	Anti-inflammatory effects via autophagy-dependent mechanisms	Xu et al., 2021 [19]
Petals	Anthocyanin-rich fraction	Inhibition of melanogenesis via ERK activation	Karunarathne et al., 2019 [20]
	Anthocyanins	Protection against oxidative stress via Nrf2/HO-1 activation	Molagoda et al., 2020 [21]
		Suppression of LPS-induced inflammation via TLR4/MD2-NFκB inhibition	Karunarathne et al., 2020 [22]
		Inhibition of NLRP3 inflammasome via NFκB and ER stress modulation	Molagoda et al., 2021 [23]
	Anthocyanin-enriched polyphenols	Anti-osteoporotic effects via GSK-3β inhibition and β-catenin activation	Karunarathne et al., 2021 [18]
		Protection against UVB-induced skin damage	Karunarathne et al., 2021 [6]
Flower	Absolute extract	Enhanced wound healing in keratinocytes	Yoon et al., 2017 [24]
	Extract (clinical trial)	Improved sleep quality in randomized controlled trial	Choi et al., 2023 [3]
Flower, leaf, root	Crude extracts	Nutritional profiling and phytochemical screening	Park et al., 2022 [5]
Flower (light purple cultivar)	Extract	Antimicrobial activity against plant pathogens	Sánchez-Hernández et al., 2021 [25]
Root bark	Ethanol extract	Antidepressant-like and neuroprotective effects	Kim et al., 2018 [26]
	Isolated triterpenoids	Cytotoxicity against cancer cell lines	Shi et al., 2014 [27]
Root and stem bark	Enzyme-treated extract	Anti-photoaging effects via increased hydration and collagen synthesis	Yang et al., 2019 [28]
Whole plant (in silico/in vivo)	Extract	Antidiabetic, antioxidant, and hepatoprotective properties	Ziyanok-Demirtas, 2024 [17]

## Data Availability

The authors confirm that the data supporting the findings of this study are available within the article.

## Abbreviations

Ang II: angiotensin II; BNP: brain natriuretic peptide; CCL20: C-C chemokine ligand 20; DCFH-DA: 2′,7′-dichlorofluorescin diacetate; ELISA: enzyme-linked immunosorbent assay; ET-1: endothelin-1; HF: heart failure; HPLC: high-performance liquid chromatography; HS: *Hibiscus syriacus* L.; HS(-Bu): butanol; HS(-EA): ethyl acetate; HS(-EtOH): ethanol; HS(-Hex): hexane; HS(-H_2_O): water; IL-1β: interleukin-1β; IL-6: interleukin-6; LPS: lipopolysaccharide; RAAS: renin–angiotensin–aldosterone system; ROS: reactive oxygen species; TNF-α: tumor necrosis factor-alpha.
